# Rolling resistance contribution to a road pavement life cycle carbon footprint analysis

**DOI:** 10.1007/s11367-016-1203-9

**Published:** 2016-10-01

**Authors:** Laura Trupia, Tony Parry, Luis C. Neves, Davide Lo Presti

**Affiliations:** Nottingham Transportation Engineering Centre (NTEC), Faculty of Engineering, University of Nottingham, University Park, Nottingham, NG7 2RD UK

**Keywords:** Carbon footprint, Greenhouse gas (GHG) emissions, LCA, Rolling resistance, Pavement surface properties

## Abstract

**Purpose:**

Although the impact of road pavement surface condition on rolling resistance has been included in the life cycle assessment (LCA) framework of several studies in the last years, there is still a high level of uncertainty concerning the methodological assumptions and the parameters that can affect the results. In order to adopt pavement carbon footprint/LCA as a decision-making tool, it is necessary to explore the impact of the chosen methods and assumptions on the LCA results.

**Methods:**

This paper provides a review of the main models describing the impact of the pavement surface properties on vehicle fuel consumption and analyses the influence of the methodological assumptions related to the rolling resistance on the LCA results. It compares the CO_2_ emissions, calculated with two different rolling resistance models existing in literature, and performs a sensitivity test on some specific input variables (pavement deterioration rate, traffic growth, and emission factors/fuel efficiency improvement).

**Results and discussion:**

The model used to calculate the impact of the pavement surface condition on fuel consumption significantly affects the LCA results. The pavement deterioration rate influences the calculation in both models, while traffic growth and fuel efficiency improvement have a limited impact on the vehicle CO_2_ emissions resulting from the pavement condition contribution to rolling resistance.

**Conclusions and recommendations:**

Existing models linking pavement condition to rolling resistance and hence vehicle emissions are not broadly applicable to the use phase of road pavement LCA and further research is necessary before a widely-used methodology can be defined. The methods of modelling and the methodological assumptions need to be transparent in the analysis of the impact of the pavement surface condition on fuel consumption, in order to be interpreted by decision makers and implemented in an LCA framework. This will be necessary before product category rules (PCR) for pavement LCA can be extended to include the use phase.

## Introduction

**Fig. 1:**
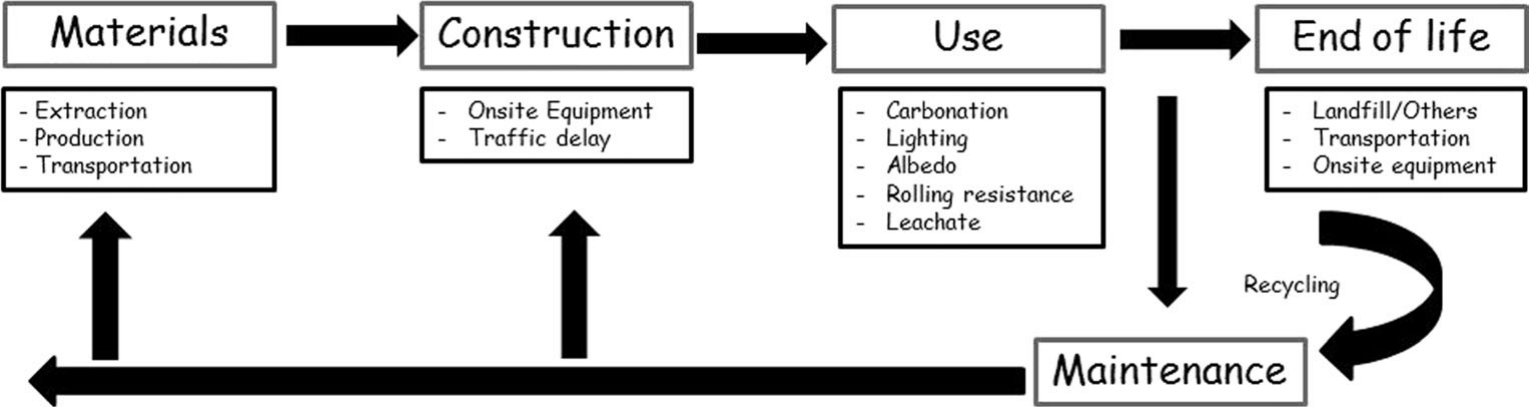
Pavement LCA framework adapted from (Santero et al. 2011b)

Road transport accounts for the majority of greenhouse gas (GHG) emissions from transport in the UK and is a significant component of all UK greenhouse gas emissions. In 2009, UK total GHG emissions from transport were 165.8 Mt carbon dioxide equivalent (CO_2_e), accounting for 27 % of total UK GHG emissions, and road transport was the most significant source of emissions, accounting for 68 % of total transport GHG emissions (UK Department of Energy & Climate Change 2015). In order to reduce this impact, in the last years, highway authorities and a growing number of organizations, companies and government institutions are introducing sustainability principles and considerations in asset management decision-making processes, by using a systematic and organized approach, called life cycle assessment (LCA) (Korre and Durucan 2009), (Wayman et al. 2014). LCA is a structured methodology to estimate and quantify the environmental impacts over the full life cycle of a product or system, “from cradle to grave”, estimating direct and indirect impacts. For pavements, a typical life cycle includes material production, construction, use, maintenance and rehabilitation (M&R), and end of life (EOL) phases (Santero et al. 2011b; Wang et al. 2014) (see Fig. 1). The use phase is one of the most critical and complex parts of a road pavement LCA, requiring specific knowledge in disparate areas (Santero and Horvath 2009). During this phase, the environmental impact is affected by several complex mechanisms; rolling resistance, albedo, carbonation, lighting and leachate. For this reason and for the uncertainty that consequently characterizes it, it is hard to quantify the impact of this phase with a sufficient level of accuracy and in the past, it was generally omitted from the framework of many LCA studies (Santero et al. 2011a), (Santero et al. 2011b). This may be acceptable for stand-alone LCA studies (e.g. to estimate the environmental impacts of a paving material) but is a problem for comparative LCA studies where different use phase outcomes could result (e.g. where different materials or maintenance programmes will lead to different surface condition) (Butt et al. 2015).

The use phase represents the longest phase in the life cycle of a pavement, remaining in service for decades (much longer than the construction phase), so it can have a significant environmental impact. In particular, it has been demonstrated that the impact of these components can span a very wide range of values (from negligible to significant), depending on different parameters (Santero and Horvath 2009). Among these components, the rolling resistance can have a dominating impact under certain conditions. The rolling resistance is the effort that the engine makes to keep the tyre rolling on the pavement. It represents the energy loss associated with the pavement-vehicle interaction (PVI), due to the physical interaction between pavement and tyre and it is mainly caused by the visco-elastic properties of the rubber elements present in the tyre tread. Although much of the rolling resistance can be tracked to tyre properties, it is also affected by other parameters related to the characteristics of the pavement, such as the pavement surface properties, macrotexture—usually represented by parameters mean profile depth (MPD) or mean texture depth (MTD)—and unevenness or pavement roughness—typically measured by the International Roughness Index (IRI). Pavement surface properties affect rolling resistance that, acting opposite to the motion of the vehicle, increases the fuel consumption. An increase in traffic fuel consumption corresponds to a growth in environmental impact, due to the increase in emission of pollutants. Therefore, vehicle energy consumption and emissions are affected by pavement surface properties; however, quantifying the influence of the pavement surface condition on the rolling resistance is complex. Over the last years, some efforts have been made to assess the overall impact of the use phase, particularly PVI. However, there is still a high level of uncertainty concerning the lack of validated models used to analyse the vehicle emissions and the influence of specific variables and assumptions on the results. In order to obtain reliable results that can be interpreted by decision makers, it is necessary that methods of modelling and the assumptions adopted in LCA and carbon footprint studies are transparent. In addition, there are no significant researches involving UK case studies on the impact of the use phase on the life cycle of a pavement. By reducing the uncertainty concerning the estimation of this component, highway authorities, research organizations and other policy-making institutions can include pavement LCA in their decision-making framework with more confidence.

This paper will analyse a UK case study to investigate the impact of extending the system boundary of road pavement LCA to include the emissions due to the effect of the pavement surface properties (IRI and MPD) on the rolling resistance. The main aim of this study is to explore if the understanding and the knowledge of this component are sufficient to be implemented in the road pavement LCA framework. The research questions are the as follows: Are rolling resistance models ready for implementation in a pavement LCA? Can they be applied to a UK case study? How do pavement deterioration and the models used to describe them affect the results?

Based on the use of two different models present in the literature, this study will estimate the range of potential impact of the rolling resistance component, with a focus on the effect of the deterioration of pavement surface condition (IRI and MPD) on traffic fuel consumption and CO_2_ emissions. By using different methodologies and making different assumptions regarding traffic growth, emission factors/fuel efficiency improvement and pavement surface condition deterioration rate, the sensitivity of the results to the different assumptions will be tested. This will allow the parameters that affect the environmental impact due to PVI rolling resistance and the magnitude of this effect to be estimated.

## Brief literature review

### Rolling resistance models

The relationship between pavement properties, rolling resistance and vehicle fuel consumption has been an area of study for several years. However, the inclusion of this component in the LCA system boundary is quite recent and is mainly focused on the potential for pavement management practice to reduce the net life cycle emissions of a road over the life cycle of well-maintained pavements. In order to define the contribution of the rolling resistance, in terms of IRI and MPD, in the use phase of a pavement LCA framework, it is necessary to use both a rolling resistance model (relating rolling resistance to pavement surface properties) and an emission model (relating traffic fuel emissions to the rolling resistance).

Starting from the 1980s, several rolling resistance measurement studies have been performed in Europe, to investigate the impact of pavement properties on rolling resistance and vehicle fuel consumption, by using different test methods (Sandberg et al. 2011b). Existing literature on the influence of road surface properties and vehicle rolling resistance, and hence emissions, presents differing results. This is due to a number of reasons: road surface contributions are a relatively small part of the driving resistance or of just the rolling resistance; it is difficult to isolate the road surface effects from other effects (i.e. tyres) and quantify the contribution of IRI and MPD; different methods of measuring rolling resistance can yield different results (Hammarström et al. 2012). Recently, different studies (Sandberg et al. 2011a; Willis et al. 2015) reviewed the most significant rolling resistance research around the world, drawing the following overall conclusions:

- When the rolling resistance coefficient increases, the vehicle fuel consumption increases significantly, especially on roads with no gradient and at constant speed (typically high highway speed) (Bendtsen 2004).
- The most significant pavement parameters affecting rolling resistance are macrotexture (MPD), or megatexture, unevenness or roughness (IRI) and stiffness.
- Texture and unevenness affect the rolling resistance in a negative way; greater values of MPD and IRI correspond to greater rolling resistance.
- For light vehicles, the impact of MPD is around three times that of the IRI effect.
- The effect of roughness on rolling resistance can change with speed, while that of texture does not.
- How stiffness affects PVI has not been consistently explained and is as yet, uncertain.

Based on these conclusions, a model describing the pavement influence on rolling resistance should take into account MPD and IRI, while the impact of stiffness is not yet clear. Pavement unevenness and macrotexture are the deviations of a pavement surface from a true planar surface with the wavelengths of deviations ranging from 0.5 to 50 m, and from 0.5 to 50 mm, respectively (International Organization for Standardization (ISO) 2004). There are few models in the literature that have explored the combined effect of IRI and MPD: Highway Development and Management Model—version 4 (HDM-4) and the model developed by the Swedish National Road and Transport Research Institute (VTI), within the European Commission project Miriam (Models for rolling resistance In Road Infrastructure Asset Management systems).

*HDM-4* is an empirical-mechanistic model software tool developed by PIARC (World Road Association) to perform cost analysis for the maintenance and rehabilitation of roads (Kerali et al. 2000)**.** It includes both a model for simulating rolling resistance from IRI and MPD and an engine model to link the effects of rolling resistance to vehicle fuel consumption**.** The mechanistic part of HDM-4 analyses all driving resistances on the engine, based on the vehicle speed and road gradient, while the empirical part uses coefficients which convert the driving resistances to energy consumption, determined through various experiments and calibrated with measured data**.** In 2011, the fuel consumption model was calibrated for US conditions as part of the NCHRP Project 1-45 (Chatti and Zaabar 2012). The results of this study showed that IRI and road gradient had a statistically significant relationship with fuel consumption at low and high speed, while macrotexture (MPD) was not statistically significant at high speed. This is contradictory to the observations of other studies, as described above. The authors explained this result by the fact that at higher speed, the air drag is the predominant component of the fuel consumption and minimizes the increase in rolling resistance due to macrotexture. In order to use HDM-4 as a road decision support tool in UK, the UK Department for Transport (DfT) and the University of Birmingham calibrated the model under English conditions (Odoki et al. 2013). Unfortunately, the calibration factors are not published.

**Fig. 2:**
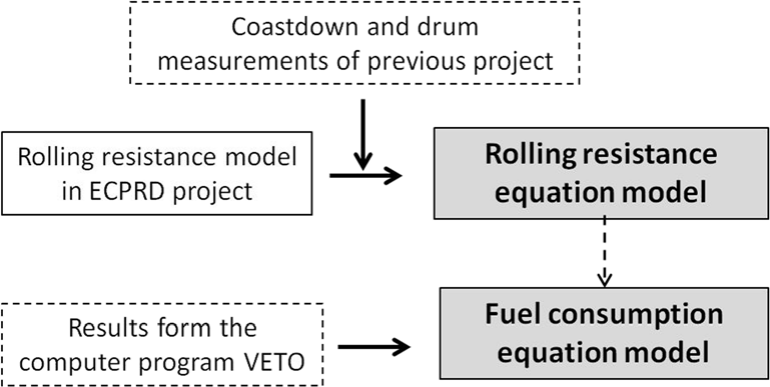
Fuel consumption model approach

The VTI model, instead (Fig. 2), includes a general rolling resistance model and a fuel consumption model; the first is mainly based on empirical data from coastdown measurements in Sweden and incorporated into a driving resistance based fuel consumption model. The fuel consumption model has been calibrated based on calculated values from the computer program VETO, a theoretical model developed at VTI to calculate fuel consumption and exhaust emissions from traffic due to various characteristics of vehicles, roads and driving behaviour (Hammarström et al. 2012). The VTI model allows the calculation of the fuel consumption related to the pavement surface properties for a car, for a heavy truck and for a heavy truck with trailer, by using two different equations: the first one relates the rolling resistance to the surface properties of a pavement (IRI and MPD) (Eq.(1)); the second one expresses the fuel consumption as a function of the rolling resistance, speed and other road condition variables, such as gradient and horizontal curvature (Eq. (2)).

Rolling resistance for a car:

1 $$ {F}_{\mathrm{r}}={m}_1\times g\times \left(0.00912+0.0000210\times IRI\times v+0.00172\times MPD\right) $$

where *m*_1_ is the vehicle mass (kg), *v* is the vehicle speed (m/s), IRI is the road roughness (m/km) and MPD is the macrotexture (mm).

Fuel consumption function for a car:

2 $$ {F}_{cs}=0.286\times \left({\left(\begin{array}{l}1.209+0.000481\times IRI\times v+0.394\times MPD\\ {}+0.000667\times {v}^2+0.0000807\times ADC\times {v}^2\\ {}-0.00611\times RF+0.000297\times {RF}^2\end{array}\right)}^{1.163}\right)\times {v}^{0.056} $$

where ADC is the average degree of curvature (rad/km) and RF is the road gradient (m/km).

### LCA studies including the rolling resistance component

As mentioned above, in the last years, some studies have started to include the impact of the pavement properties in the pavement LCA framework. Table 1 summarizes the major LCA studies, which include the effect of pavement surface condition on rolling resistance within the system boundary. The table shows that overall, there are just a few recent studies including the effect of both roughness and texture and they use the HDM-4 or the VTI models, described above.

**Table 1:** Relevant pavement LCA studies including the effect of the use phase

Study	Country	Rolling resistance components included	Comments
Santero and Horvath (2009)	USA	Roughness	Rough estimation based on literature data
Zhang et al. (2009)	USA	Roughness	Simple linear relationship between IRI and fuel consumption based on data from heavy duty trucks only, tested at low speed on test track
Wang et al. (2012b)	USA	Roughness and texture	HDM-4 was used to consider the rolling resistance and MOVES (Motor Vehicle Emission Simulator) (EPA’s Office of Transportation and Air Quality (OTAQ) 2014) was used to model the vehicle emissions as a function of rolling resistance
Yang (2014)	USA	Roughness	Model presented by Zaabar and Chatti (2010)
Santos et al. (2015)	Portugual	Roughness and texture	Model presented by Hammarström et al. (2012)
Bryce et al. (2014)	USA	Roughness and texture	Model presented by Hammarström et al. (2012) and from the National Cooperative Highway Research Program (NCHRP) (Chatti and Zaabar 2012)
Araújo et al. (2014)	Portugual	–	The energy consumption variation associated with different rolling resistances of the surface layers is evaluated with laboratory tests
Wang et al. (2014)	USA	Roughness and texture	The vehicle CO_2_ emission factors are estimated as a continuous function of MPD and IRI, by using HDM-4 and MOVES (Motor Vehicle Emission Simulator)
Xu et al. (2015)	USA	Roughness	Model presented by Zaabar and Chatti (2010)

**Fig. 3:**
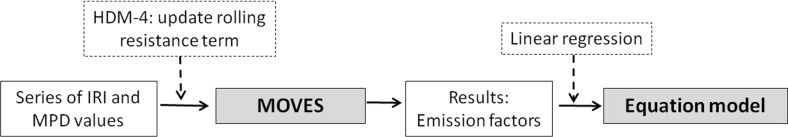
Emission model approach

An interesting approach is the one developed by Wang et al. (Wang et al. 2012b, 2014) at the University of California Pavement Research Center (UCPRC, Davis). In this model (Fig. 3), HDM-4 was used to estimate the rolling resistance and MOVES (Motor Vehicle Emission Simulator) (EPA’s Office of Transportation and Air Quality (OTAQ) 2014) was used to model the vehicle emissions as a function of rolling resistance. In order to develop the equation function, the authors have modelled a series of IRI and MPD values for combinations of specific variables (pavement type, road type, road access type, vehicle type mix) using MOVES. The estimated emission factors depend on different variables, including the tyre rolling resistance represented by a default coefficient. This default value has been obtained through dynamometer tests on a smooth surface (usually steel or steel with a sand coating) and therefore, it only takes into account the influence of the tyre on the rolling resistance, neglecting the effect of the pavement properties. In order to calculate the emissions under different IRI and MPD conditions, the default rolling resistance coefficient has been updated in the MOVES database by using the formula adopted in the HDM-4 software that also includes the effect of the pavement properties on the rolling resistance (Wang et al. 2012a).

The model developed with this approach is shown in Eq.(3):

3 $$ {T}_{CO_2}={a}_1\times MPD+{a}_2\times IRI+\mathrm{Intercept} $$

where ${T}_{CO_2}$is the tailpipe CO_2_ emission factor; the terms *a*_1_, *a*_2_ and Intercept are the coefficients derived from the linear regression, depending on surface type and access type, year and vehicle type; IRI is the road roughness (m/km) and MPD is the macrotexture (mm). In particular, the Intercept term represents the CO_2_ emissions due to the total driving resistance, except the contribution of the pavement deterioration, estimated with the other two components.

### Parameters affecting the results of the rolling resistance component in LCA studies

The use of all these models, correlating pavement surface properties to vehicle fuel consumption and emissions, requires the estimation of some parameters that can affect the final result, including the pavement condition deterioration rate with time (in terms of IRI and MPD), the traffic growth and the emission factors/fuel efficiency improvements.

During the use phase of a road pavement, pavement deterioration leads to changes in unevenness and macrotexture that vary over time based on different variables, pavement material (asphalt or concrete), traffic volume and truck traffic, climate, pavement age and maintenance treatments (Wang et al. 2014). Roughness (IRI) tends to increase over time for a specific road but the variation of the texture depth (MPD) can be positive or negative, depending on several mechanisms. Unlike in the USA for instance, in the UK, new surfaces are generally produced with high initial texture depth to maintain high-speed skidding resistance and a reduction in texture depth over time is observed, especially in the more trafficked lanes. The rate of reduction depends on several variables; for instance, after a surface dressing, the embedment of chippings into the underlying layer, under the action of traffic, produces a rapid drop in the texture depth over the first 1 or 2 years. The final value that the texture depth reaches depends on the substrate of the surface dressing and the size of aggregate used for chippings. Other surfacing materials, like rolled asphalt, do not change so markedly during the first few years, but the average texture tends to reduce in subsequent years, at least in the more trafficked lanes (Jacobs 1982), (UK Goverment 1999). This type of behaviour has also been observed in other studies related to other European countries (Hammarström et al. 2012). Several studies have been performed in the UK in order to predict performance in terms of texture depth. In addition, the UK Roads Board has developed SCANNER (Surface Condition Assessment for the National Network of Roads) surveys, to provide a consistent method of measuring the surface condition (including ride quality, rut depth, intensity of cracking, texture depth and edge condition) (Transport Research Laboratory 2009). However, there are no general models in the UK able to predict the change of texture depth over time.

**Fig. 4:**
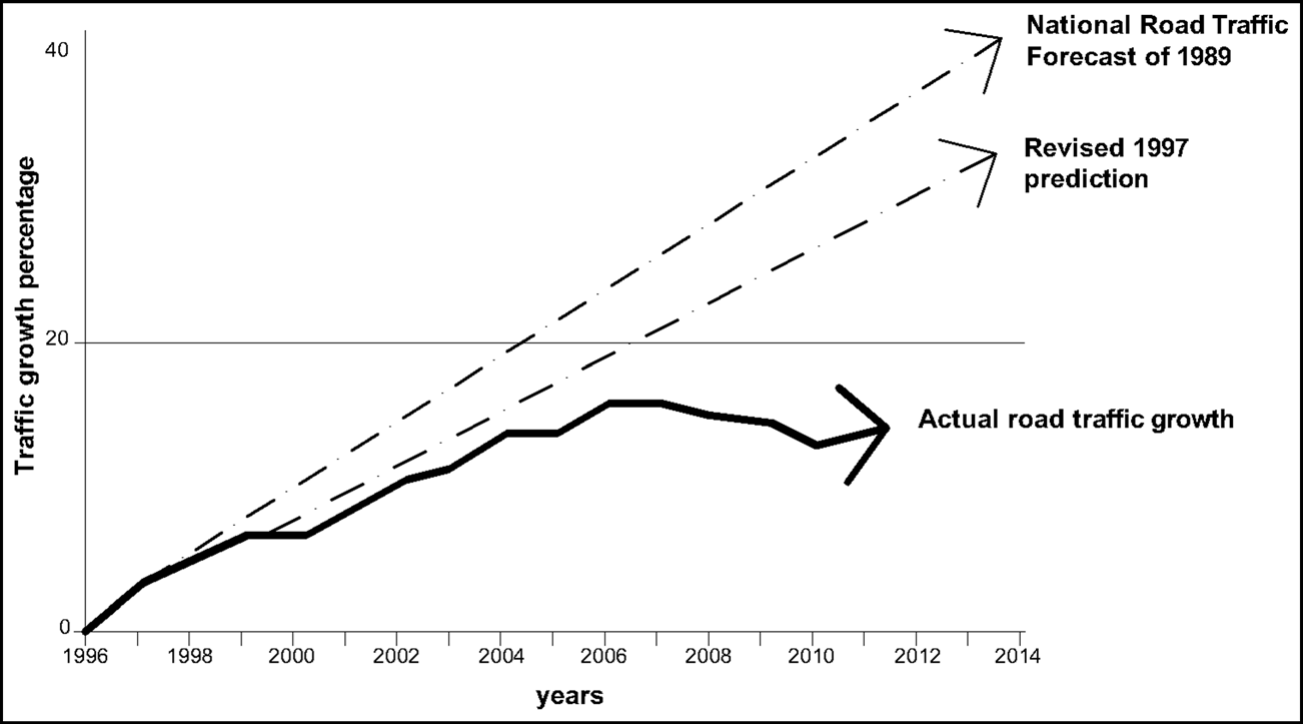
Predictions and actual traffic growth in the UK (readapted from (Masters 2015))

Another important variable necessary to quantify the future level of traffic emissions is the traffic growth factor. It requires the understanding of how people make travel choices and the expected path of key drivers of travel demand. Recent studies (Masters 2015) have shown how in the UK the rates of traffic growth are consistently overestimated by the Department for Transport (see Fig. 4) and traffic congestion is a limiting factor for large traffic growth, so this parameter is an uncertain factor that could significantly impact the results. Finally, the emission factors and fuel consumption or efficiency improvements should be taken into account, in order to estimate future levels of emissions. This estimation is particularly complex, since it requires the prediction of future technological improvements, based on the announced government policy. In the UK, the Department for Transport’s National Transport Model (NTM) has provided forecasts of CO_2_ emission changes by vehicle type between 2010 and 2040, taking into account technological improvements in fuel type and efficiency (UK Department for Transport 2013a).

Since a high level of uncertainty characterizes this area of knowledge, both in terms of available models and in terms of parameters affecting the results, this study aims to investigate the impact of the pavement surface properties (IRI and MPD) during the use phase, in terms of CO_2_ emissions, by using two different models in the literature (VTI and UCPRC model) and performing a sensitivity analysis to investigate the variables and conditions that can have an impact.

### Case study

The case study analysed in this paper is a 720-m section of road—200-m length of dual carriageway (typical width, 22 m) and 520-m length of single carriageway (typical width, 11 m)—located in Lincolnshire on the A17 between Sutton Bridge and Kings Lynn, an inter-urban road in the UK East Midlands. The annual average daily flow (AADF) in 2009 was 15,372 motor vehicles and 2412 HGVs, making this segment a low to medium trafficked road. The existence of previous studies focusing on the construction and maintenance phases of this road segment (Galatioto et al. 2015; Huang et al. 2013; Spray 2014) is one of the main reasons why it was chosen as a case study. This will allow a better understanding of the relative environmental impact and the magnitude of the use phase, in terms of rolling resistance impact, on the LCA of this case study, by comparing it with the construction and maintenance results. In addition, there is an appropriate level of information and data available on the history of construction and maintenance events and on the traffic flow, provided by Lincolnshire Highways Authority. Lastly, based on the UK road type classification, this is an ‘A’ road, a major road intended to provide large-scale transport links within or between areas (UK Department for Transport 2012). Motorways and major trunk A roads account for a small percentage of the UK road network in length, but they carry a large and consistently increasing amount of traffic. In 2014, major roads combined accounted for 13 % (1 % motorway and 12 % ‘A’ roads) of road length and carried 65 % of total road traffic in Great Britain (21 % motorway and 45 % ‘A’ roads), as has been the case over the past 10 years. (UK Department for Transport 2016).

The original construction of this road segment dates back to 1989 followed by some minor maintenance treatments until 2009, when a major rehabilitation took place. The full depth reconstruction involved milling out of 150 mm of the old asphalt pavement and replacing with an inlay of new asphalt mixtures and the use of a proprietary reinforcing Gridseal system (composite asphalt reinforcement system (CRS)). The analysis period chosen for this case study is 20 years, starting in 2009 until 2029 when a future rehabilitation is assumed. Short analysis periods are more reliable in terms of predictions (e.g. traffic growth, vehicle technology evolution, maintenance strategies, etc.), since evolving performance expectations and demand create a high level of uncertainty over longer analysis periods. In other research involving this same case study, the impact of raw materials, construction and maintenance (but not traffic delay) phases have been investigated (Spray 2014), giving an estimate of 370 tCO_2_e for the 2009 reconstruction. In addition, a recent study, including the traffic emission’s impact due to delays during maintenance works in 2009 (Galatioto et al. 2015), concluded that the impact of this component can span between 1.94 and 16.46 t of CO_2_ (the greatest component of the vehicle tailpipe CO_2_e), depending on the traffic flow and the maintenance strategy adopted (Table 2).

**Table 2:** Results of previous studies on the A17 case study

Results for the base case scenario	
LCA phase	Tonne
2009 reconstruction	370 CO_2_e
2009 traffic delay for the work-zone	1.94–16.46 CO_2_
2009–2029 use phase (rolling resistance due to pavement surface MPD and IRI)	This paper

## Methodology

**Fig. 5:**
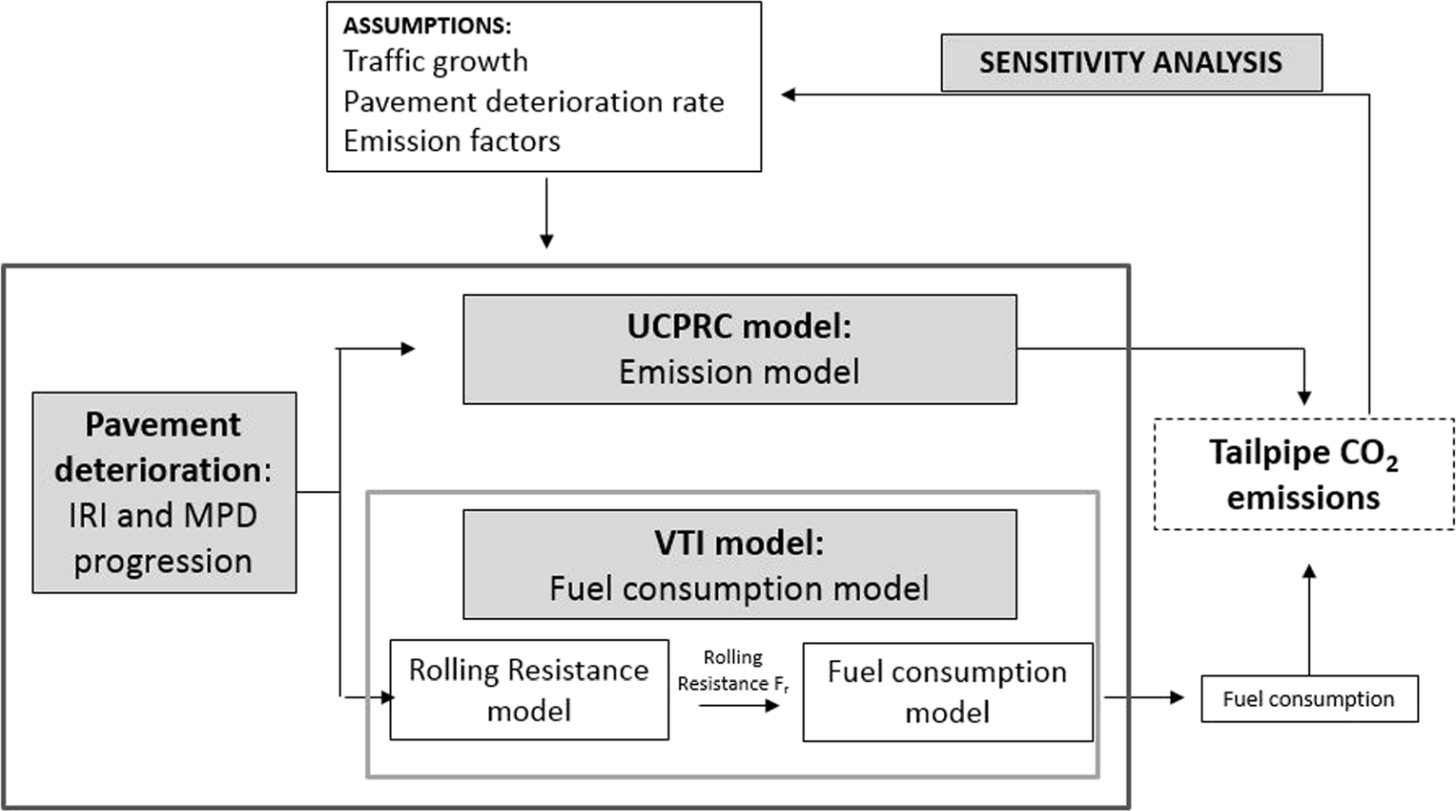
Outline of the process adopted in this study

This study will estimate the additional GHG emissions from vehicle operation due to pavement surface properties and their deterioration for the case study section, by using two different models developed in literature (VTI and UCPRC models) and will conduct a sensitivity analysis on some factors influencing the results (traffic growth, pavement deterioration model and emission factors/fuel efficiency improvements). Since CO_2_ is the greatest component of the vehicle tailpipe CO_2_e emissions (over 99.8 %) (Wang et al. 2014), other tailpipe emissions are not taken into account in this study. Figure 5 shows the outline of the process adopted.

### Calculation of the tailpipe CO_2_ emissions with VTI and UCPRC model

First, the time progression of pavement surface deterioration (IRI and MPD) is generated, according to literature data for specific M&R strategies (Jacobs 1982; UK Goverment 1999), (Aavik et al. 2013; Wang et al. 2014). In order to estimate the range of potential impact of the pavement deterioration during the use phase, different scenarios of deterioration of IRI and MPD for the same road segment are considered.

**Fig. 6:**
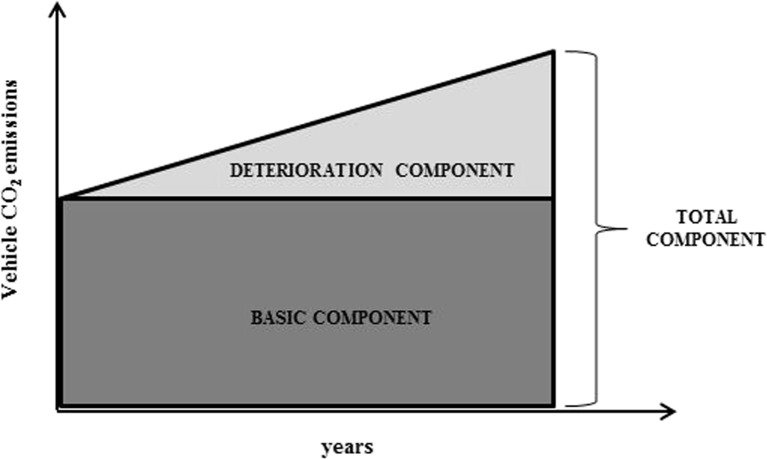
Total CO_2_ emissions, divided into basic (*dark grey area*) and deterioration components (*light grey area*), for a case without traffic growth and emission factor change

In the UCPRC model, the vehicle CO_2_ emission factors are estimated as a continuous function of MPD and IRI (Wang et al. 2014). The CO_2_ emission factors for a specific vehicle type are calculated directly, based on the analysed pavement segment’s MPD and IRI by using Eq. (3) and multiplying by the vehicle mileage travelled. The VTI model includes a general rolling resistance model (Eq. (1)) and a fuel consumption model (Eq. (2)) so that it is possible to estimate the contribution of the rolling resistance to the total driving resistance and hence vehicle fuel consumption (Hammarström et al. 2012) and then to convert it to CO_2_ emissions, assuming the conversion process proposed by the International Carbon Bank & Exchange (ICBE) (2010). Since this paper is focused on estimating the impact of the pavement surface properties (IRI, MPD) that affect rolling resistance at the pavement—vehicle interface, for both models, only the CO_2_ emissions directly related to these elements are taken into account in the results (the other terms of the equations are considered equal to zero). The two models allow the estimation of the total CO_2_ emissions related to the pavement condition in terms of IRI and MPD (see Fig. 6), namely the total component (total area, representing the total CO_2_ emissions related to the IRI and MPD), including the basic component (dark grey area, representing the value of emissions if the IRI and MPD remain constant over time—no deterioration) and the deterioration component (light grey area, equal to the difference between the first two and representing the emissions due to the deterioration of the pavement properties, in terms of IRI and MPD).

The deterioration component is particularly interesting for pavement engineering, since it is possible to reduce these emissions associated with the road surface condition, through appropriate maintenance strategies. Pavement condition improvements can be obtained rapidly to reduce traffic fuel consumption, even using available technology. On the other hand, approaches that involve technology improvements or traffic reductions can require long periods of time. In order to better understand the behaviour of the two PVI emission models and the impact of the pavement deterioration assumptions, these components were assessed in a sensitivity analysis. Once the results in terms of CO_2_ emissions are obtained from both models, it is possible to compare them, in order to understand the potential impact of the model used on the pavement LCA results. Furthermore, in order to identify the parameters that most affect results in the use phase, a sensitivity analysis is performed for the following variables: traffic growth, IRI and MPD time progression and vehicle fuel emission factors.

### Sensitivity test

- Traffic growth model

The AADF data for this study is extracted from the traffic dataset provided by the UK Department for Transport (UK Department for Transport 2014), where the vehicle data is classified based on the area, the year and the vehicle type. In order to quantify the impact of pavement surface properties on the use phase, it is necessary to estimate the future AADF, using a growth factor. This was estimated using TEMPRO (Trip End Model Presentation Program) (UK Department for Transport 2013b), a tool developed by the UK Department for Transport that analyses local data and, used in conjunction with national or regional traffic growth forecasts, provides local traffic projection factors. Since traffic growth is an uncertain factor, the sensitivity test performed for this variable took into account three different scenarios: the first one includes the estimated traffic growth projections (average), the second assumes no traffic growth during the analysis period (no), and the third one, a further increase of the traffic growth projections of 10 % (average + 10 %). The traffic growth factor was assumed to evolve linearly over the lifetime of the pavement.

- Pavement deterioration

In the literature, there are some empirical models calibrated for specific areas and maintenance treatments, to describe the deterioration rate of IRI and MPD. However, these models are site specific and not applicable to this case study (in these models, the value of MPD tends to increase over time, which is not typical in the UK). Since the focus of this study is on estimating the range of potential impact, the time progression of IRI and MPD on the assessed road segment over the analysis period (20 years) is generated according to literature data for specific M&R strategies (Aavik et al. 2013; Jacobs 1982; UK Goverment 1999; Wang et al. 2014) and by taking into account the following scenarios:

- ‘average’ deterioration scenario (IRI increases from 1.0 to 2.3 m/km and MPD decreases from 1.8 to 0.8 mm);
- ‘worst’ deterioration scenario (IRI increases from 1.0 to 5.0 m/km and MPD is 1.5 mm during all the analysis period).
- ‘no deterioration’ scenario where the surface pavement condition is unchanged over time (IRI = 1.0 m/km and MPD = 1.5 mm).

- Emission factor or fuel efficiency improvement

In order to test the sensitivity of the main inputs to the two models, different scenarios of variation of the emission factors in the UCPRC model and fuel efficiency in the VTI model will be considered. In the UCPRC model, changing the emission factors based on the MOVES software (that result in the coefficients a_1_, a_2_ and Intercept of the linear regression, developed in Wang et al. (2014)) will be assessed. These factors change year by year based on predictions of future fuel economy and new vehicle technologies (e.g. electric vehicles). In the VTI model, changing the fuel efficiency will be tested, by using road emission projections resulting from the Department for Transport’s National Transport Model (NTM) (UK Department for Transport 2013a). Again, in order to assess the sensitivity of the results to the emission factor forecast, three different scenarios are considered over the analysis period: emission factors and fuel efficiency constant (no); emission factors reduction and fuel efficiency increase, based on MOVES and NTM projections (average); and further variation of 10 % in emission factors reduction and fuel efficiency increase based on MOVES and NTM projections (average + 10 %).

Based on the different assumptions made for the traffic growth, pavement deterioration and emission factors/fuel efficiency, different cases are analysed and compared. The traffic growth and the pavement deterioration during the analysis period tend to increase the CO_2_ emissions, while the emission factor reduction affects the results in the opposite way, as vehicles become more fuel efficient.

## Results

In order to evaluate the results, two baseline case scenarios have been defined (Table 3): the base case scenario to compare the results from two rolling resistance models and the average case scenario to compare the results of the sensitivity test (based on the different assumptions made for the traffic growth, pavement deterioration and emission factors/fuel efficiency).

**Table 3:** Base and average case scenario parameters

Case scenario	Pavement deterioration	Traffic growth	Fuel efficiency /emission factors	Comments
Base case scenario	Average	No	No	Comparison of rolling resistance models
Average case scenario	Average	Average	Average	Comparison of sensitivity test

### Comparison of the CO_2_ emissions calculated with the VTI and UCPRC models

**Fig. 7:**
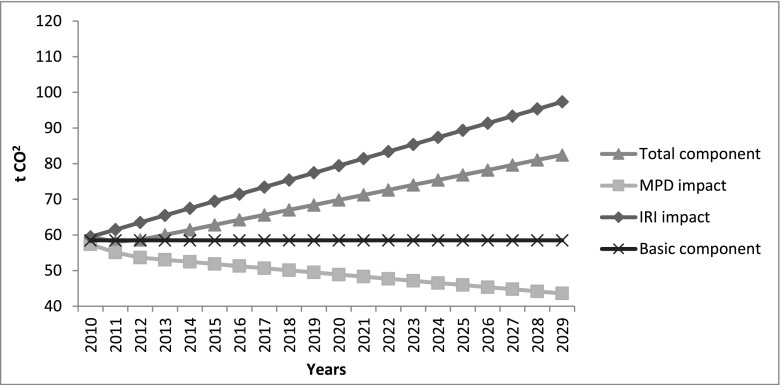
Impact of IRI and MPD in the UCPRC model

**Fig. 8:**
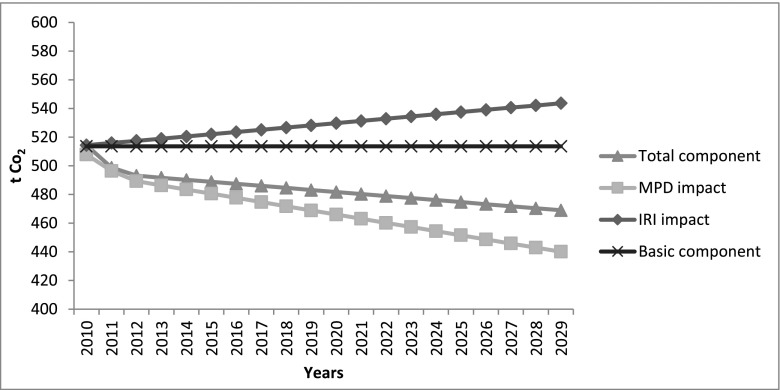
Impact of IRI and MPD in the VTI model

Table 4 summarizes the life cycle CO_2_ emissions for the pavement case study analysed. As already described, since CO_2_ is over 99.8 % of the vehicle tailpipe CO_2_e emissions, other tailpipe emissions are not taken into account for the traffic delay and for the use phase. For the use phase, the table shows the results obtained by using the two rolling resistance models, taking into account the total emissions and the deterioration component of the base case scenario (no traffic growth, no emission factor changes and average pavement deterioration). The UCPRC results show that, overall, the impact of the pavement surface properties on the life cycle of the case study—compared to the construction phase—is significantly higher, if the total emissions are considered (1387 tCO_2_ vs 370 tCO_2_e), and of the same order of magnitude, if only the deterioration is considered (217 tCO_2_ vs 370 tCO_2_e). In the VTI model, on the other hand, the total emissions are more than one order of magnitude higher than the construction phase (9672 tCO_2_ vs 370 tCO_2_e) and the deterioration component is a negative term (−600 tCO_2_). Clearly, the two models provide considerably different results, both in terms of the general contribution of the pavement surface properties to the rolling resistance (basic component) and in terms of the impact of the different components (IRI and MPD). These differences are due to different factors. The two models were calibrated for different countries, by using different background data (weather, vehicles, and roads) and they use two different approaches. The UCPRC model yields directly the PVI CO_2_ emissions related to a specific pavement type, road type (and speed), road access type and vehicle type mix. The coefficients in the model take into account improvements in vehicle technology and the reduction of the emission factors over time. In the VTI model, instead, it is possible to calculate the fuel consumption related to a specific type of vehicle at a specific speed (and the IRI term is directly correlated to the speed). This requires the conversion of the fuel consumption into CO_2_ emissions, by using specific conversion factors for fuel that do not take into account the vehicle age and technology. The negative term related to the deterioration component is a result of the different weight given to the IRI and MPD terms (see Fig. 7 and Fig. 8). In the VTI model, even at high speed (that increases the impact of the IRI), the MPD term has a larger impact on the emission estimate. In the UCPRC model, the IRI term has a larger impact. This difference has a large impact on results for pavement surfaces where the IRI tends to increase and the MPD tends to decrease, as for this case study. In the VTI model, the MPD term tends to decrease faster than the IRI term increases, providing a negative result for the deterioration component. The results show that the choice of model used to estimate the CO_2_ emissions related to the pavement surface properties and the deterioration model are instrumental, since the different models give very different results.

**Table 4:** Results for the base case scenario

Results for the base case scenario				
LCA phase			Result	
2009 reconstruction			370 tCO_2_e	
2009 traffic delay for the work-zone			1.94–16.46 tCO_2_	
		Total emissions	Basic component	Deterioration component
2009–2029 use phase (rolling resistance due to pavement surface MPD and IRI)	UCPRC model	1387 tCO_2_	1170 tCO_2_	217 tCO_2_
	VTI model	9672 tCO_2_	10,272 tCO_2_	−600 tCO_2_

### Sensitivity analysis

Table 5 and Table 6 show the results of the sensitivity tests for the variables: traffic growth, IRI and MPD deterioration rate, emission factors or fuel efficiency. By evaluating the best and the worst case scenarios for the two different models and considering the impact on the basic, deterioration and total components of vehicle CO_2_ emissions, the sensitivity analysis shows the following:

- for both models, the potential emissions due to PVI rolling resistance have a large range of values;
- this is particularly so in the deterioration component, especially in the VTI model, where the CO_2_ emissions can vary between 0.80 and 7.38 times the average value;
- the best case scenario (lowest emissions) occurs under different assumptions for the two models (no deterioration in the UCPRC model and average deterioration in the VTI model). In the UCPRC model, the deterioration component increases over time, so the absence of deterioration minimizes the total emissions. In the VTI model, the deterioration component, under the average condition of pavement deterioration, tends to decrease, producing an overall reduction in the calculated emissions. This effect levels off under the ‘worst deterioration’ pavement condition, when the IRI effect is larger than the MPD effect.

**Fig. 9:**
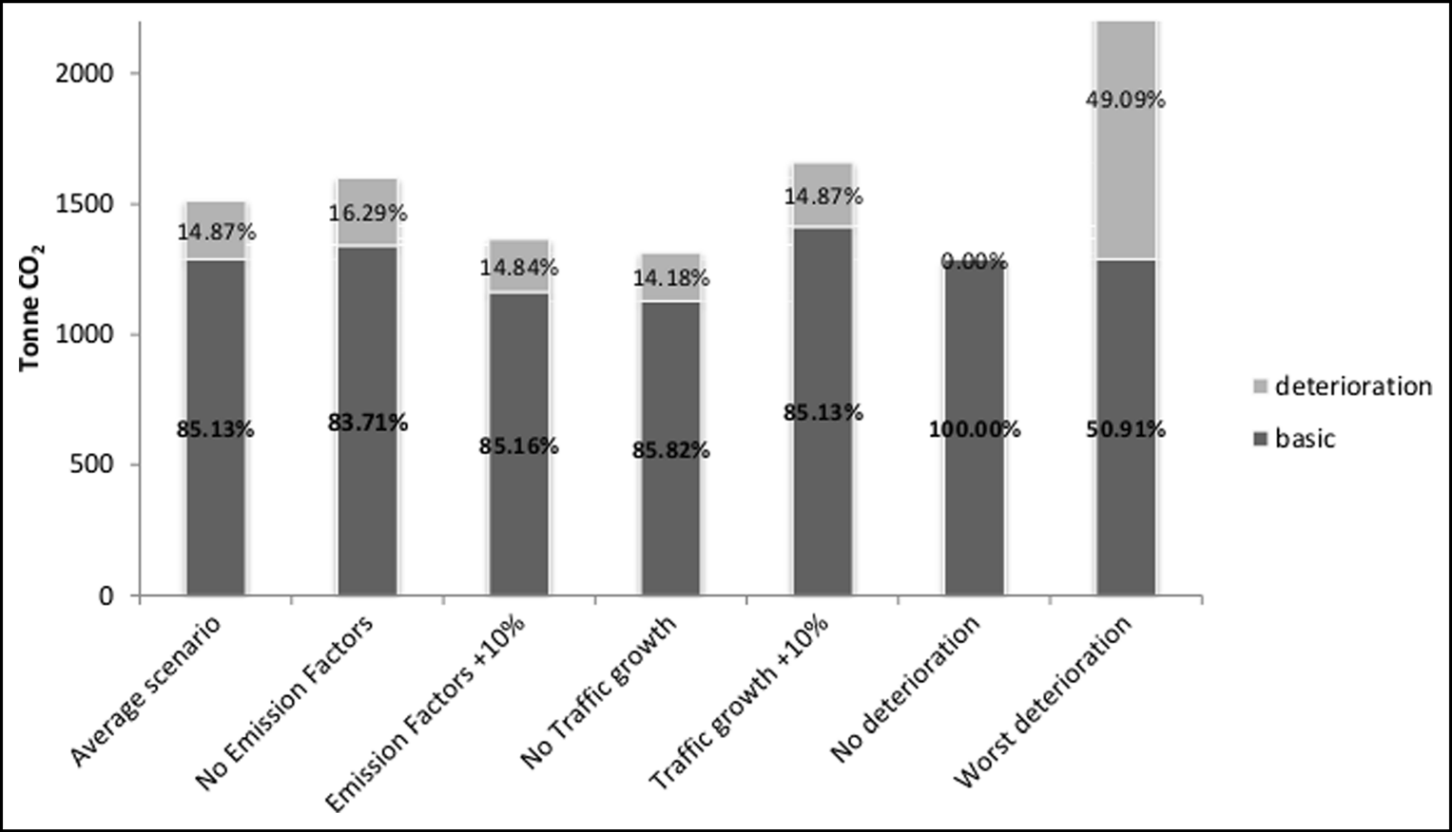
Sensitivity analysis—impact of each variable on emissions due to pavement rolling resistance (UCPRC model)

**Fig. 10:**
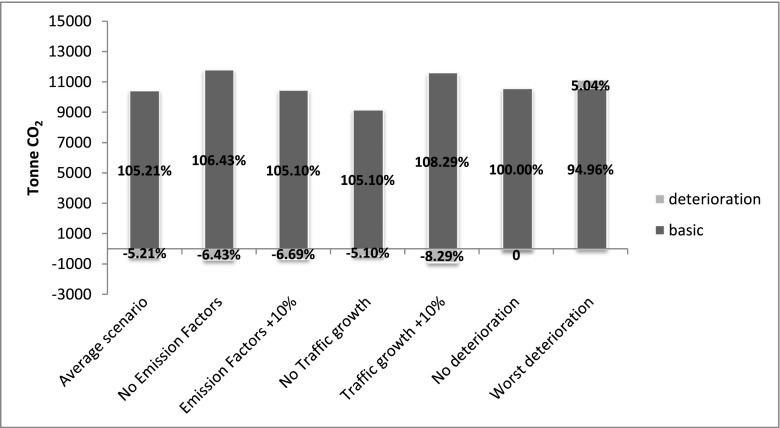
Sensitivity analysis—impact of each variable on emissions due to pavement rolling resistance (VTI model)

To better understand the impact of the different variables, Fig. 9 and Fig. 10 show a comparison between the average case scenario and six other scenarios where, in their turn, only one parameter is changed between its minimum and maximum value. The deterioration component remains between 14 and 16 %—in the UCPRC model—and between −5 and −8 %—in the VTI model—of the total component in each case, with the exception of the worst deterioration scenario, especially in the UCPRC model (around 50 % of the total component). This implies that the traffic growth and the emission factors/fuel economy changes do not significantly affect the results, either in terms of the basic component or in terms of the deterioration component, at least for this case study (only in the VTI model does a large increase of the traffic level produce a moderate impact on emissions). This is because while the traffic growth during the analysis period tends to increase the CO_2_ emissions, the emission factor reduction affects the results in the opposite way, as vehicles become more fuel efficient. Therefore, even if the traffic growth and the emission factor parameters affect the results, this combined impact is not significant overall. By contrast, the CO_2_ emissions due to the pavement roughness are very sensitive to the pavement surface deterioration over time.

In both models, the CO_2_ emissions are significantly higher in the case of the worst pavement deterioration scenario. This result agrees with other works (Araújo et al. 2014; Wang et al. 2012a, 2014) that show how optimized maintenance strategies aimed at reducing pavement deterioration over time and the use of suitable materials can have a significant influence on vehicle CO_2_ emissions during the use phase of a pavement.

**Table 5:** Sensitivity test results for the UCPRC model

Case scenario	Sensitivity parameter			Emission of CO_2_ (tonne)		
	Pavement deterioration	Traffic growth	Emission factors	Basic	Deterioration	Total
Average case scenario	Average deterioration	Average	Average	1288	225	1513
Best case scenario	No pavement deterioration	No	Average + 10 %	(−21 %) 1020	(−100 %) 0	(−33 %) 1020
Worst case scenario	Worst deterioration	Average + 10 %	No	(+36 %) 1755	(+438 %) 1210	(+96 %) 2965

**Table 6:** Sensitivity test results for the VTI model

Case scenario	Sensitivity parameter			Emission of CO_2_ (tonne)		
	Pavement deterioration	Traffic growth	Emission factors	Basic	Deterioration	Total
Average case scenario	Average deterioration	Average	Average	10,372	−514	9858
Best case scenario	Average deterioration	No	Average + 10 %	(−12 %) 9141	(+8 %) −557	(13 %) 8584
Worst case scenario	Worst deterioration	Average + 10 %	No	(−1 %) 10,272	(−738 %) 3281	(+37 %) 13,553

## Discussion

Based on the use of two different models, this paper assessed the impact of pavement surface deterioration during the use phase of a specific UK road pavement case study, with the objective of estimating the overall impact of this component on life cycle CO_2_ emissions and the parameters that can affect the results. This makes it possible to define future research needs in this area and to understand the level of confidence possible in decision making using pavement LCA results. The results agree with previous studies in the literature (Santero and Horvath 2009; Wang et al. 2012b)**,** showing that the pavement surface properties (IRI and MPD) have a significant impact during the life cycle of the pavement, compared to the other phases (370 t of CO_2_ for the reconstruction phase and between 1.94 and 16.46 t of CO_2_ for the traffic delay phase); the CO_2_ emissions related to this component are significant both for the deterioration component (between −600 and 217 t of CO_2_) and the total component (between 1170 and 10,272 t of CO_2_).

However, the results obtained using the two models are significantly different, both considering the basic component of emissions due to PVI rolling resistance (not affected by the pavement surface deterioration) and the deterioration component. These considerable differences are due to the fact that the development of rolling resistance and fuel consumption models is strongly affected by methodological components (such as different rolling resistance measuring methods, road surface measures, approach used to develop the models) and by site-specific components (weather, vehicle types and technology, type of roads, pavement design models and deterioration). The UCPRC model was developed in California, using the HDM-4 model calibrated for US conditions and MOVES, the US EPA highway vehicle emission model based on national data. The VTI model developed in Sweden includes a rolling resistance model based on empirical data and a fuel consumption model calibrated using calculated values from VETO, a theoretical model. California and Sweden are geographical locations characterized by different climates, types of roads, pavement deterioration processes and models, traffic distribution and technology, that seriously affect the models developed and the results produced. The two models consider the impact of the pavement surface properties, IRI and MPD, in different ways. In the UCPRC model, the IRI has a larger impact on the rolling resistance than the MPD and the opposite consideration is true for the VTI model. This difference is particularly significant in this case study, where the MPD falls over time, producing opposite results when the two models are used; in the UCPRC model, the deterioration component is positive, since the impact of the increase in IRI is larger than that due to the reduction in MPD, while for the VTI model, the deterioration component is negative. Therefore, the pavement condition deterioration over time has a strong impact on the rolling resistance, significantly affecting the results. This is confirmed by the sensitivity test performed on the IRI and MPD deterioration rate that showed that the CO_2_ emissions due to PVI rolling resistance are very sensitive to this factor. By contrast, in this case study with low to medium traffic, traffic growth and the emission factors/fuel economy changes do not have a large impact on the results, because they tend to offset each other.

Butt et al. (2015) discuss the use of attributional and consequential LCA studies for road pavements, where environmental impacts are attributed to products or actions, or the consequences or relative changes of making different decisions are estimated, respectively. These types of study can be used to estimate impacts in stand-alone studies of a single material or process, or in comparative studies of different choices. The two models used in this study use different approaches, described above, and this results in significantly different findings, which reduces confidence in their use for all types of LCA study, which will all be sensitive to the model chosen.

Traffic growth and future changes in vehicle fuel efficiency and fuel types can be expected to have a significant impact on future emissions from road transport. Current predictions for the UK mean that these factors offset each other and combine to have little effect on the results for this case study. This means that the results of UK pavement LCA studies are not very sensitive to these factors. However, considering one factor without the other will distort the results and changes in the forecasts for these factors need to be monitored and studies updated to reflect them.

The potential impact of the factors explored in this study on the results of pavement LCA including the use phase is significant. For this reason, LCA practitioners should be careful to report the models and assumptions they use in a detailed and transparent way (Huang and Parry 2014). Development of widely accepted approaches and agreement to use and declare them is a prerequisite for the development of LCA practice in this domain.

## Conclusions and recommendations

The main aim of this paper was to investigate if current models of the impact of pavement surface properties on rolling resistance can be implemented in road pavement LCA. Considering the significant impact that the pavement surface properties can have during the life cycle of a road, it is necessary that any model used to estimate this component leads to results that can be used with confidence in the decision-making process. Taking into account the results obtained in the selected case study, the use of the UCPRC and VTI models in the UK should be treated with caution because they produce significantly different results. Further and different case studies are needed before it can be decided where they can be used. The different weight that the models give to the different pavement condition variables means the relative results from the two models are very sensitive to both level of pavement condition and its deterioration rate. This will have an impact both on stand-alone and comparative LCA studies.

For UK roads, there is currently insufficient information available to predict the deterioration of roughness and texture depth over time depending on maintenance treatments, traffic volume, surface properties and materials. This must be corrected before pavement LCA studies can be extended to the use phase. Traffic growth and the emission factors/fuel efficiency predictions, combined to predict future vehicle emissions, have a relatively small effect because they cancel out to a large extent. Changes in predicted future traffic levels or emission factors could change this result and should be kept under review.

Further research is necessary before the effect of the rolling resistance can be introduced in the pavement LCA framework with confidence. In particular, for UK roads, research is needed to develop reliable pavement deterioration models and PVI rolling resistance models, before introducing this component. LCA and carbon footprint studies need to be reported in a way that makes the methods of modelling and the assumptions used transparent, before they can be interpreted by decision makers. Standard models and procedures should be developed in the pavement LCA field to make this possible and are needed before product category rules in this domain can be extended to include the use phase.
